# 3′-UTR Shortening Contributes to Subtype-Specific Cancer Growth by Breaking Stable ceRNA Crosstalk of Housekeeping Genes

**DOI:** 10.3389/fbioe.2020.00334

**Published:** 2020-04-29

**Authors:** Zhenjiang Fan, Soyeon Kim, Yulong Bai, Brenda Diergaarde, Hyun Jung Park

**Affiliations:** ^1^Department of Computer Science, University of Pittsburgh, Pittsburgh, PA, United States; ^2^Department of Pediatrics, School of Medicine, University of Pittsburgh, Pittsburgh, PA, United States; ^3^Division of Pulmonary Medicine, Children's Hospital of Pittsburgh UPMC, Pittsburgh, PA, United States; ^4^Department of Human Genetics, Graduate School of Public Health, University of Pittsburgh, Pittsburgh, PA, United States; ^5^Hillman Cancer Center, University of Pittsburgh Medical Cancer, Pittsburgh, PA, United States

**Keywords:** competing-endogenous RNA, housekeeping genes, breast cancer subtypes, RNA regulation, alternative polyadenylation (APA)

## Abstract

Shortening of 3′UTRs (3′US) through alternative polyadenylation is a post-transcriptional mechanism that regulates the expression of hundreds of genes in human cancers. In breast cancer, different subtypes of tumor samples, such as estrogen receptor positive and negative (ER+ and ER–), are characterized by distinct molecular mechanisms, suggesting possible differences in the post-transcriptional regulation between the subtype tumors. In this study, based on the profound tumorigenic role of 3′US interacting with competing-endogenous RNA (ceRNA) network (3′US-ceRNA effect), we hypothesize that the 3′US-ceRNA effect drives subtype-specific tumor growth. However, we found that the subtypes are available in different sample sizes, biasing the ceRNA network size and disabling the fair comparison of the 3′US-ceRNA effect. Using normalized Laplacian matrix eigenvalue distribution, we addressed this bias and built tumor ceRNA networks comparable between the subtypes. Based on the comparison, we identified a novel role of housekeeping (HK) genes as stable and strong miRNA sponges (sponge HK genes) that synchronize the ceRNA networks of normal samples (adjacent to ER+ and ER– tumor samples). We further found that distinct 3′US events in the ER- tumor break the stable sponge effect of HK genes in a subtype-specific fashion, especially in association with the aggressive and metastatic phenotypes. Knockdown of NUDT21 further suggested the role of 3′US-ceRNA effect in repressing HK genes for tumor growth. In this study, we identified 3′US-ceRNA effect on the sponge HK genes for subtype-specific growth of ER- tumors.

## Introduction

Approximately, 70% of human genes contain multiple polyadenylation (polyA) sites in the 3′-untranslated region (3′-UTR) (Mayr and Bartel, 2009). Through alternative polyadenylation (APA) during transcription, messenger RNAs (mRNA) from the same gene can have various 3′-UTR lengths. Since the 3′-UTR contains regulatory regions including microRNA (miRNA) target sites, mRNAs with shortened or lengthened 3′-UTRs may diversify the regulation landscape, for example, miRNA binding landscape. In human cancer, 3′-UTR lengthening (3′UL) has been associated with cell senescence (Chen M. et al., 2018) with implications for tumor-associated processes, such as cell cycle inhibition, DNA damage/repair process, and tumor suppression (Dimri et al., 1995; Busuttil et al., 2003; López-Otín et al., 2013; Muñoz-Espín and Serrano, 2014). Widespread 3′-UTR shortening (3′US) has been reported for diverse types of human cancer (Mayr and Bartel, 2009). Furthermore, 3′US events add prognostic power beyond common clinical and molecular covariates in cancer patients (Xia et al., 2014) and are associated with drug sensitivity in cancer cell lines (Xiang et al., 2018). These results suggest that APA events, both 3′-UTR shortening and lengthening, play important roles in cancer etiology and treatments.

The 3′-UTR is also implicated in competing-endogenous RNA crosstalk (ceRNA) (Salmena et al., 2011). ceRNAs co-regulate each other's RNAs through competing for binding miRNAs. In diverse types of cancer, ceRNA regulation involves established oncogenes and tumor-suppressor genes (Sumazin et al., 2011) and facilitates molecular pathway interactions for tumorigenesis (Park et al., 2018a). When 3′-UTR shortening genes lose miRNA target sites on their 3′-UTRs and do not sequester the miRNAs, the associated miRNAs bind to the 3′-UTR of the ceRNA partners. As a result, 3′-UTR shortening disrupts ceRNA crosstalk (3′US-ceRNA effect) for growth in diverse types of cancer, including breast cancer (Park et al., 2018b). In a recent study, we showed that this 3′US-ceRNA effect promotes tumor growth independent of potential confounding factors, such as somatic mutation status (SNPs and small INDELs), tumor purity, immune cell infiltration, cell proliferation, or miRNA biogenesis and expression (Kim et al., 2019).

Breast cancer can be classified into two major subtypes based on the presence or absence of estrogen receptor (ER) (Hammond et al., 2010). Estrogen receptor positive (ER+) breast tumors grow in the presence of the hormone estrogen. So, ER+ cancers can be treated with endocrine therapy which blocks ER activity or depletes estrogen levels. On the other hand, estrogen receptor negative (ER-) breast tumors have a unique growth mechanism due to absence of the estrogen receptor. The unique growth mechanism of ER- tumors makes it difficult to treat ER- breast cancer that has a worse prognosis than ER+ (Tsutsui et al., 2002) with a more aggressive phenotype (Sheikh et al., 1994; Perou et al., 2002). Based on the profound tumorigenic effect of 3′US-ceRNA (Park et al., 2018b), we hypothesize that 3′US-ceRNA effects specific to ER- breast tumors contribute to the unique growth mechanism. In this study, we tested this hypothesis by addressing a quantitative challenge due to the different sample sizes between ER+ and ER- breast tumor samples. As a result, we identified a novel subset of housekeeping (HK) genes (sponge HK) effectively sponging miRNAs to synchronize the ceRNA networks in normal samples (adjacent to the subtype tumor samples). Furthermore, we showed that the 3′US-ceRNA effects repress the sponge HK genes, leading to subtype-specific tumor growth. In ER- breast tumor, this subtype-specific tumor growth is associated with aggressive and metastatic phenotypes of ER- tumors, attributing its unique growth mechanism partially to subtype-specific 3′US-ceRNA effects.

## Materials and Methods

### TCGA Breast Tumor RNA-seq Data and Identification of Breast Cancer Subtypes

Quantified gene expression files (RNASeqV1) for primary breast tumors (TCGA sample code 01) and their matching solid normal samples (TCGA sample code 11) were downloaded from the TCGA Data Portal (Goldman et al., 2013). We used 97 breast tumor samples that have matched normal tissues, which were further categorized into 77 estrogen receptor positive (ER+) and 20 estrogen receptor negative (ER–). For ER+ and ER–, we collected both normal (ER+ normal and ER- normal) and tumor (ER+ tumor and ER- tumor) samples. A total of 10,868 expressed RefSeq genes [fragments per kilobase of transcript per million mapped reads (FPM) ≥1 in >80% of all samples] were selected for downstream analyses.

### Selection of miRNA Target Sites

The predicted miRNA target sites were obtained from TargetScanHuman version 6.2 (Lewis et al., 2005). Only those with a preferentially conserved targeting score of more than 0 were used (Xia et al., 2014). Experimentally validated miRNA target sites were obtained from TarBase version 5.0 (Papadopoulos et al., 2009), miRecords version 4 (Xiao et al., 2009), and miRTarBase version 4.5 (Hsu et al., 2014). The target sites found in indirect studies such as microarray experiments and high-throughput proteomics measurements were filtered out (Dvinge et al., 2013). Another source is the microRNA target atlas composed of public AGO-CLIP data (Hamilton et al., 2013) with significant target sites (*q* < 0.05). The predicted and validated target site information was then combined for use in this study. Among 1,261 miRNAs curated in the TCGA BRCA data, we used 713 expressed ones (average FPM >1) in our analyses (Supplementary Table 7).

### Statistical Significance of Pearson Correlation Coefficient

The implementation of the Pearson r function is provided by a python package, SciPy, and available at https://scipy.org/, which returns the calculated correlation coefficient and a two-tailed *p*-value for testing the non-correlation. The Pearson correlation coefficient measures the linear relationship between two variables (e.g., gene X and gene Y), and when the two covariates follow a binormal distribution, we can assume that their Pearson's correlation follows Student's *t*-distribution. The *p*-value is calculated by three steps: (1) calculating the value of the Pearson's correlation *t*, (2) defining the degree of freedom *df* (*N*-2, where *N* is the sample size), and (3) getting the probability of having *t* or more extreme than *t* from a Student's *t*-distribution with the degrees of freedom *df*. We used hypergeometric test in Scipy to estimate the significance of miRNA binding site overlap between genes.

### Detection of APA Events

We used DaPars (Xia et al., 2014) to identify 3′UTR shortening and lengthening in RNA-Seq data based on the same cutoff and parameter values optimized in the original paper. We checked that our prediction is 100% matched with that of the original DaPars result. The DaPars paper provided multiple lines of evidence to demonstrate that DaPars indeed identified APA events in the TCGA data. First, 51% of the DaPars predictions are within 50 bp of the annotated APAs compiled from Refseq, ENSEMBL, UCSC gene models, and polyA database [(polyA_DB Lee et al., 2007]. Second, in the upstream (−50 nt) of the predicted APA sites, MEME motif enrichment analysis (Bailey et al., 2009) successfully identified canonical polyA signal AATAAA.

### Housekeeping, Transcription Factor, and Tumor-Associated Genes

Housekeeping genes are required for the maintenance of basic cellular functions that are essential for the existence of a cell, regardless of its specific role in the tissue or organism. Generally, housekeeping (HK) genes are expected to be expressed at relatively constant rates in most non-pathological situations (Eisenberg and Levanon, 2003). We used 3,804 HK genes defined in RNA-Seq data for 16 normal human tissue types: adrenal, adipose, brain, breast, colon, heart, kidney, liver, lung, lymph, ovary, prostate, skeletal muscle, testes, thyroid, and white blood cells (Eisenberg and Levanon, 2013).

Transcription factors (TFs) play an important role in the gene regulatory network. We downloaded 2,020 TF genes defined in the TFcheckpoint database (Chawla et al., 2013), in which TF information is collected from nine different resources. Among them, we used 1,020 genes that are further supported by sequence-specific DNA-binding RNA polymerase II activity.

The tumor-suppressor genes and oncogenes were defined by the TUSON algorithm from genome sequencing of >8,200 tumor/normal pairs (Davoli et al., 2013), in particular, residue-specific activating mutations for oncogenes and discrete inactivating mutations for tumor-suppressor genes. TUSON computationally analyzes patterns of mutation in tumors and predicts the likelihood that any individual gene functions as a tumor-suppressor gene or oncogene. We used 466 oncogenes and 466 tumor-suppressor genes at the top 500 in each prediction (after subtracting 34 genes in common).

### Building Subtype ceRNA Networks

For each of the breast cancer data (ER+ normal, ER+ tumor, ER- normal, and ER- tumor) that we defined above, we constructed a ceRNA network based on the microRNA (miRNA) target site share and expression correlation (Ala et al., 2013; Park et al., 2018b). The same miRNA target site information was determined regardless of the subtypes, resulting into the miRNA target site share network (based on FDR > 0.05 in hypergeometric test with miRNA target site information). Given the same miRNA target site share network, the expression correlation information for each subtype will select ceRNA network edges for each subtype.

We first constructed the ER+ normal reference ceRNA network by applying a traditional correlation cutoff (≥0.6) on the miRNA target site share network. Then, to identify ER- normal ceRNA network comparable to ER+ normal reference ceRNA network, we applied different correlation cutoff values (0–1, with a step size of 0.01) on the miRNA target site share network for ER- normal samples and selected the correlation cutoff values that make ER- normal ceRNA network most similar to the ER+ normal reference ceRNA network. To estimate topological similarity, we employed normalized Laplacian matrix eigenvalue distribution that discovers ensembles of Erdos–Rényi graphs better than other metrics such as Sequential Adjacency or Laplacian (Gera et al., 2018). After identifying the ER+ normal reference network and the corresponding ER- normal network, we used the same cutoffs (0.6 for ER+ subtypes and 0.68 for ER- subtypes) to construct the ER+ tumor network and the ER- tumor network, respectively. An overall workflow is presented in Supplementary Figure 1.

### Estimating Topological Similarity

To identify the structural equivalence between two networks, we employed spectral analysis not only to identify the structural similarities but also to track down the underlying dynamic behavior changes between them. Spectral clustering on networks uses the eigenvalues of several matrices, such as adjacency matrix, the Laplacian matrix, and the normalized Laplacian matrix. In this research, we used the normalized Laplacian matrix since it involves both the degree matrix and adjacency matrix, where the degree matrix can identify the node-related equivalence of networks and the adjacency matrix can capture the structural equivalence of networks. Another very important reason of using the normalized Laplacian eigenvalue matrix is that it is more sensitive to small changes because it considers more information (Perou et al., 2002).

For network G, the normalized Laplacian of G is the matrix:

(1) $$\begin{array}{r} N=D^{-1/2}-LD^{-1/2} \end{array}$$

where L is the Laplacian matrix of G and D is the degree matrix. The Laplacian matrix L is defined as: *L* = *D* − *A*, where A is the adjacency matrix of G.

In N, each of its entry elements is given by:

(2) $$\begin{array}{r} N_{i,j}=\{\begin{array}{cc} 1,\; & if\;i=j\;and\text{ degree}\left(v_{i}\right)\neq 0\\ -\frac{1}{\sqrt{\mathrm{degree}\left(v_{i}\right)\text{degree}\left(v_{j}\right)}},\; & if\;i\neq j\;and\;v_{i}\;is\;adjacent\;to\;v_{j}\\ 0,\; & otherwise \end{array} \end{array}$$

where degree (vertex v) is the function that returns the degree of the vertex v.

To assess how close the two networks G_1_ and G_2_ are, we first built N1 and N2 based on the connection information of G1 and G2, respectively. Then, we defined *dist*_1_ and *dist*_2_ as the eigenvalue distribution of N1 and N2, respectively. We further used the Kolmogorov–Smirnov test (KS test), which is defined as:

(3) $$\begin{array}{r} K_{1,2}=\;\underset{x}{\text{sup}}|dist_{1}\left(x\right)-dist_{2}\left(x\right)| \end{array}$$

where $\sup_{x}$ is the supremum of the set of distances.

By using the normalized Laplacian matrix and KS test, ER+ normal reference network $G_{ref}^{ER+}$ is compared with ER- normal subnetwork with a particular correlation cutoff *i* $G_{i}^{ER-\;}$in the following three steps:

- Compute the normalized Laplacian metrics $N_{ref}^{ER+\;}$ and $N_{i}^{ER-\;}$ from $G_{ref}^{ER+\;}$ and $G_{i}^{ER-\;}$, respectively.
- Compute the eigenvalues $E_{ref}^{ER+\;}$ and $E_{i}^{ER-\;}$ from $N_{ref}^{ER+\;}$ and $N_{i}^{ER-\;}$, respectively.
- Compute the KS statistic between $E_{ref}^{ER+\;}$ and $E_{i}^{ER-\;}$.

The third step tests the null hypothesis that eigenvalues $E_{ref}^{ER+\;}$ and $E_{i}^{ER-\;}$ are drawn from the same continuous distribution. If the two-tailed *p*-value returned by the KS test is high, then we cannot reject the hypothesis that $G_{ref}^{ER+\;}$ and $G_{i}^{ER-\;}$ are the same network. In another word, the higher the *p*-value is, the more similar are $G_{ref}^{ER+\;}$ and $G_{i}^{ER-\;}$.

## Results

### Widespread 3′-UTR Shortening and Lengthening Events for ER+ and ER–

**Figure 1:**
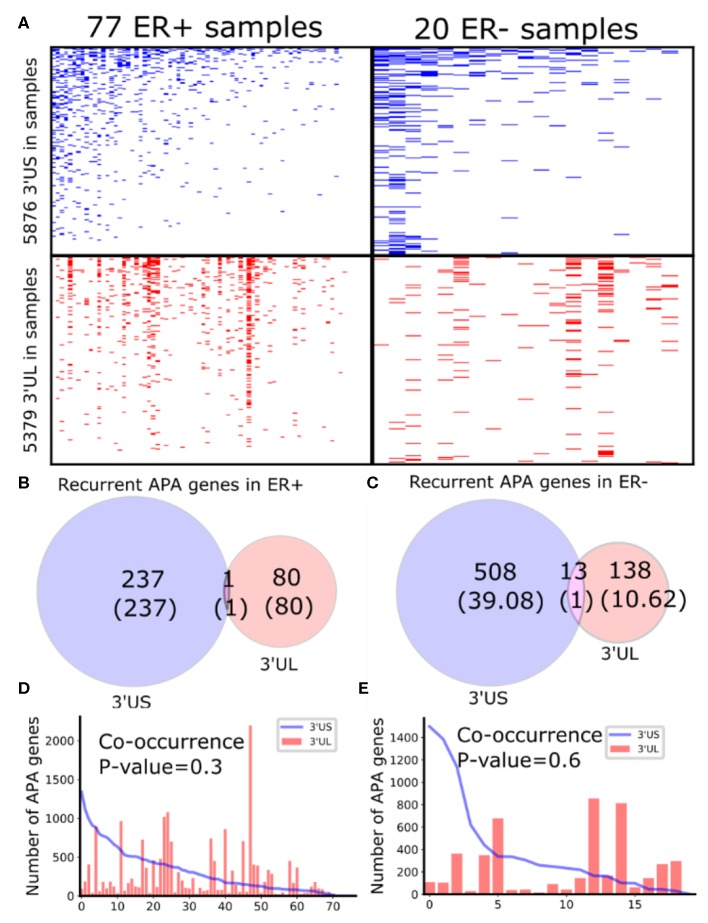
Global APA events distinct for ER+ and ER-. **(A)** Heatmaps showing the genes with 3′US (top panel) or 3′UL (bottom panel) in ER+ samples (left column) or ER- samples (right column), ranked by the total number of APA events. **(B,C)** Overlap of the recurring (>20% in samples) 3′US and 3′UL genes in ER+ and ER-, respectively. **(D,E)** The number of APA genes (3′US in line and 3′UL in red bar) in the tumor–normal sample pairs in ER+ and ER-, respectively, ordered as in **(A)**.

To identify subtype-specific APA genes, we first identified 77 ER-positive (ER+) and 20 ER– negative (ER–) sample pairs (breast tumor and the adjacent normal samples) from 97 sample pairs available in TCGA (see “MATERIALS AND METHODS”). Then, we identified 3′UTR shortened (3′US) and 3′UTR lengthened (3′UL) genes (tumor vs. normal) using DaPars (Xia et al., 2014) in each subtype. We found that the ER+ and ER- sample pairs have similar numbers of total 3′US genes and 3′UL genes (Figure 1A). However, the 3′US genes are more recurrent [occurring in >20% of the tumor samples (Xia et al., 2014)] in both subtype tumors (Figures 1B,C; e.g., *P* = 5.0 × 10^−5^ for ER+). Further analyses showed that 3′US and 3′UL play distinct roles in the subtypes. First, the recurrent 3′US and 3′UL genes show little overlap (one and 13 genes in common, *P* = 1.27e^−6^ and *P* = 3.97e^−9^, respectively; Figures 1B,C). Second, the number of 3′UL events is not correlated with that of 3′US events across the tumor samples (*P* = 0.35 for ER+ and *P* = 0.61 for ER–; Figures 1D,E). Third, ingenuity pathway analysis (IPA) shows that the recurrent 3′US and 3′UL genes are enriched for distinct sets of molecular pathways (Supplementary Table 1, Supplementary Figure 2). The IPA analysis further suggests that the 3′UL or 3′US genes themselves have limited roles for cancer overall since a small number of pathways are significantly (*P* < 10^−2^) enriched for them (12 and 14 for 3′UL in ER± and 29 and three for 3′US in ER± samples) and at most a couple of them are “cancer” pathways (one for 3′UL in ER+ and two for 3′US in ER- with keyword “cancer”). Based on the profound tumorigenic role of 3′US in its interaction with ceRNAs (3′US-ceRNA effect) (Park et al., 2018b), we hypothesize that 3′US-ceRNA effect, not 3′US cis effect, promotes ER- specific tumor growth.

### Two-Step Pairwise Normalization of ER+ and ER-ceRNA Network

We previously identified the 3′US-ceRNA effect in the ceRNA network (Park et al., 2018b). To identify the 3′US-ceRNA effect specific to ER- tumors, we aim to build ceRNA networks for ER- and ER+ tumors and compare them. Computationally, the ceRNA gene pairs in the networks are those that share a significant number of miRNA target sites and are co-expressed (Ala et al., 2013; Park et al., 2018b). However, using the common co-expression cutoff (e.g., Pearson's ρ > 0.6) will inflate the number of edges for ER- (160,687 in ER- normal vs. 88,275 in ER+ normal; Supplementary Figure 3A). To test if this inflation is attributable to the sample size difference, we built the ceRNA network 100 times from different numbers of (20, 40, 60, and 75) normal subsamples from ER+ tumors based on the same co-expression cutoff (Supplementary Figure 3B). In general, the number of edges in the ceRNA networks increases as the subsample size decreases. Especially when the same number of samples (20) to that of the ER- normal network is used, the number of edges in the subsampled networks becomes closer to the case of the ER- normal network.

Since the network size difference is attributable to the sample size difference, one might want to subsample ER+ normal samples to match the number of samples for ER- (*n* = 20). To assess this solution, we subsampled 20 ER+ normal samples 100 times, built a ceRNA network for each subsample, and collected all the edges (916,999) across the networks. Then, we checked how many times each edge occurs across the 100 subsampled networks. We found that the subsampled ceRNA networks do not keep topological consistency within them as 22.1% (202,997) of the edges are shared by <20 ceRNA networks (Supplementary Figure 3C). Then, one might want to build the ER- ceRNA network using the co-expression cutoff with the same statistical significance to ER+ (0.91, *P* ~ = 10^−8.2^; Supplementary Figure 3D). To achieve the same statistical significance of the traditional cutoff value (0.6) of ER+, the cutoff value of ER- would inflate to 0.91, resulting in a drastically deflated number of edges (Supplementary Figure 3D). We addressed this issue in the following way. First, we built the reference network from normal samples of larger size (ER+) using the common correlation cutoff (Pearson's ρ > 0.6). Since the normal samples should have similar molecular dynamics between ER+ and ER-, we sought to find the co-expression cutoff for the ER- normal network that yields the most topological similarity to the ER+ reference network. To estimate topological similarity, we employed a normalized Laplacian matrix eigenvalue distribution that discovers ensembles of Erdos–Rényi graphs better than other metrics, such as Sequential Adjacency or Laplacian (Gera et al., 2018) (see “MATERIALS AND METHODS”). While ER- normal network topology changes drastically if different correlation cutoff values are used (Supplementary Figures 3E,F), we found that the cutoff of 0.68 makes the ER- normal network most similar to the ER+ reference network (Supplementary Figure 3G). Using another measure for topological similarity, average clustering coefficient (Friedel and Zimmer, 2006), the cutoff of 0.68 is supported again since normal ER- network with correlation cutoff 0.68 makes the closest average clustering coefficient to the reference network (0.4; Supplementary Figure 3H). Since normal and tumor ceRNA networks within each subtype share the same number of samples and thus would not suffer from this bias (Altay et al., 2011; Chen H. et al., 2018; Park et al., 2018b; Dalgiç et al., 2019), we applied the subtype-specific cutoffs (0.68 for ER- and 0.6 for ER+) to build the tumor ceRNA networks in each subtype.

### 3′UTR Shortening Is Associated With the Aggressive Metastatic Phenotypes of ER– Tumors in ceRNA

**Figure 2:**
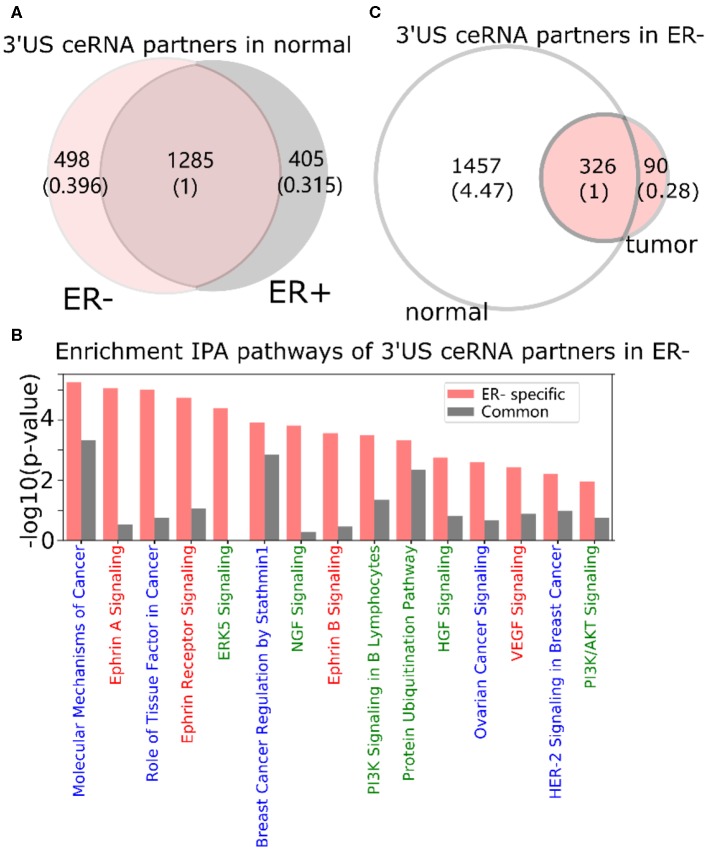
3′UTR shortening is associated to ER-'s aggressive phenotypes in ceRNA. **(A)** Intersection of 3′US ceRNA partners between ER- and ER+ normal ceRNA networks. **(B)** IPA canonical pathways significantly (*P* < 0.01) enriched for the ER- 3′US ceRNAs. The pathways are color-coded by keyword: “cancer” in blue, “signaling” in red, and those associated with aggressive phenotypes (Krishnan et al., 2013) in green. **(C)** Intersection of 3′US ceRNA partners in ER- between normal and tumor ceRNA networks.

In a normal ER- ceRNA network based on the subtype-specific co-expression cutoff, 1,783 genes are in the ceRNA relationship with 521 3′US genes (3′US ceRNA partners). Among 1,783 3′US ceRNA partners, 498 (27.9%) are found only in ER- (ER- 3′US ceRNA partners), whereas the other 1,285 (72.1%) are also in ER+ as 3′US ceRNA partners (common 3′US ceRNA partners; Figure 2A). We found that 118 IPA canonical pathways significantly (*P* < 0.01) enriched for the ER- 3′US ceRNA partners (Supplementary Table 2) are linked with several aspects of ER- specific tumor phenotypes (Figure 2B). The first set of the pathways are “cancer” pathways. For example, the “molecular mechanisms of cancer” pathway (*P* = 10^−5.25^) includes a comprehensive set of genes, disruptions of which are known to promote tumor growth. Specific to breast cancer, the enrichment of the “breast cancer regulation by Stathmin1” (*P* = 10^−3.92^) pathway is interesting since the overexpression of Stathmin1 correlates with loss of the ER (Curmi et al., 2000) and with aggressive breast tumor phenotypes (Obayashi et al., 2017). The second category of pathways underlies the aggressive metastasis of ER- tumors. For example, among eight pathways that were shown to play roles in breast tumor metastasis (Krishnan et al., 2013), we found that five of them are significantly enriched for ER- 3′US ceRNA partners with the exception of PI3K/AKT, the enriched p-value of which is just below the significance cutoff (*P* = 10^−1.95^). Furthermore, previous studies have associated breast tumor malignancy and poor survival with the abnormal control of Ephrin A [reviewed in (Vaught et al., 2008)], which is strongly enriched for ER- 3′US ceRNA partners (*P*-value = 10^−5.05^). In normal samples without 3′-UTR shortening, 3′US ceRNA partners should closely regulate these pathways. However, in ER- tumors characterized by widespread 3′US events, most (81.7%) of the 3′US ceRNA partners lost the ceRNA relationship (Figure 2C), likely losing the normal control.

### Housekeeping Genes Keep ER+ and ER- Normal ceRNA Networks to Similar Topology

**Figure 3:**
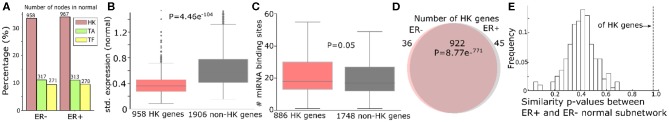
Housekeeping genes make consistent ceRNA networks between ER- and ER+ normal samples. **(A)** Number (and the percentage to the total number of nodes in the networks) of housekeeping (HK), tumor-associated (TA), or transcription factor (TF) genes in the ER- and ER+ normal ceRNA networks. **(B)** Standard deviation of gene expressions of 958 HK genes and 1,906 non-HK genes in the ER- normal ceRNA network. **(C)** Number of miRNA binding sites on the 3′UTR of 886 HK and 1,748 non-HK genes in the network. **(D)** Number of HK genes shared by ER- and ER+ normal ceRNA networks. **(E)** Distribution of the similarity of *p*-values between the subnetworks of ER+ and ER- normal ceRNA networks with 922 HK genes or the same number of non-HK genes. The higher the *p*-value is, the more similar the networks are Gera et al. (2018).

**Figure 4:**
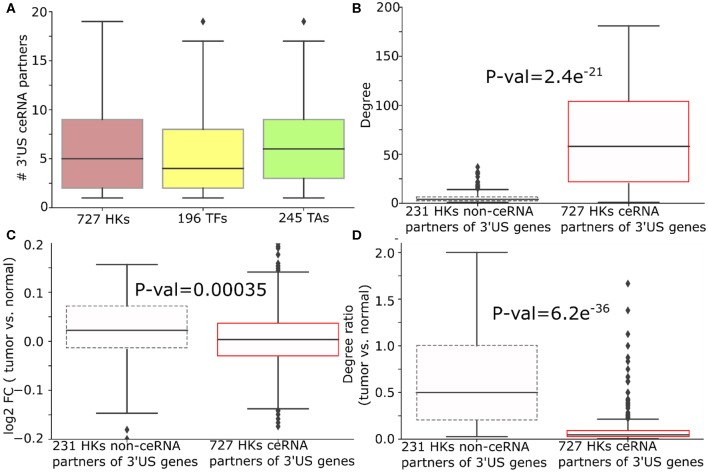
3′US disrupts ceRNA relationship of HK genes in ER- tumors. **(A)** Number of 3′US genes connected to housekeeping (HK), transcription factor, and tumor-associated genes in the ER- ceRNA network. **(B)** Degree (number of neighbors in ER- normal ceRNA network), **(C)** log_2_ fold change (tumor vs. normal), and **(D)** degree ratio (tumor vs. normal) of 727 and 231 HK genes that are ceRNA partners of 3′US genes or not, respectively. The degree ratio in **(D)** represents the ratio of the number of neighbors retained in the tumor.

Furthermore, we categorized genes that have a possible sponge effect (>5 miRNA binding sites in the 3′UTR) into housekeeping genes, tumor-associated genes (tumor suppressors or oncogenes; TA), and transcription factors. Based on 3,804 HK (Eisenberg and Levanon, 2013), 932 TA (Davoli et al., 2013), and 1,020 TF genes (Chawla et al., 2013) curated in public databases (see “MATERIALS AND METHODS”), the ceRNA networks consist of threefold more HK genes than TA or TF genes (Figure 3A for normal and Supplementary Figure 4A for tumor). Due to their active roles in cell maintenance (Eisenberg and Levanon, 2013), HK genes are expected to maintain constant expression levels under most physiological conditions (Eisenberg and Levanon, 2013). Accordingly, the 958 HK ceRNA genes in ER- normal (Figure 3A) express as highly as (Supplementary Figure 4B), but with less significant variation (*P* = 1.72e^−54^) across the normal samples (Figure 3B), the 1,906 non-HK ceRNA genes in the network. With our observation that the HK genes contain more miRNA binding sites than the other genes (*P* = 0.05, Figure 4C), they should function as stable sponges for miRNAs (Tay et al., 2014). Thus, with a significant number (*P* = 8.77e^−771^) of overlap in the HK ceRNA genes between ER- and ER+ normal samples (Figure 3D), we hypothesize that they keep ER- and ER+ normal ceRNA networks in similar topology. To test this hypothesis, we first selected edges involving the HK ceRNA genes from the ER+ and the ER- normal ceRNA networks to form subnetworks and compared the subnetworks using normalized Laplacian matrix eigenvalue distribution. Furthermore, we randomly subsampled the same number of edges not involving HK genes 200 times from the ER+ and ER- ceRNA networks and compared the networks in the same way (Figure 3E). The HK ceRNA networks are significantly more similar between ER+ and ER- (*P* < 0.01) than the 200 non-HK ceRNA networks, suggesting that HK genes make normal ceRNA crosstalk consistent between the subtypes through the miRNA sponge effect.

### 3′US Disrupts ceRNA Crosstalk of Housekeeping Genes for ER- Specific Growth

We further examined the impact of 3′US on the role of HK genes. First, 3′US genes are highly connected to HK genes. Out of 958 HK genes, 727 HK genes (75.8%) are connected to 3′US genes, which is in the same scale as the other classes of genes that are known to be regulated by 3′US genes (Sumazin et al., 2011; Park et al., 2018b) [196 (61.8%) TA genes and 245 (90.2%) TF genes; Figure 4A]. Also, these HK genes are more highly connected in the network compared to 231 HK genes that are not connected to 3′US genes (Figure 4B). Previously, we showed that 3′US represses the ceRNA partners in tumor (Park et al., 2018b). Consistently, these HK genes, ceRNA partners of 10.2 3′US genes on average (Supplementary Table 5), are more repressed in tumor than the 231 HK genes not connected to 3′US genes (*P* = 0.00035; Figure 4C). For example, transforming growth factor beta regulator 1 (TBRG1) is connected to four 3′US genes (PPP6C, DICER1, H2AFV, and UBL3) in ER- normal samples. With 3′US in ER- tumor samples, TBRG1 is significantly down-regulated (log fold change = −0.15) considering the general up-regulation of the other housekeeping genes (Figure 4C). TBRG1 and those four 3′US genes are predicted to share binding sites of miR-874 (see “MATERIALS AND METHODS”). MiR-874 was experimentally shown to repress TBRG1 to promote non-familial breast cancer (Bastos et al., 2014). Although miR-874 was expressed (average FPM is 5.3 and 5.1 in ER- tumor and normal samples), they were not significantly (*P* = 0.58) up-regulated in ER- tumor samples to repress TBRG1. The 3'UTR shortening of the four genes may instead redirect miR-874 to bind more efficiently on TBRG1, leading to its repression. We checked that TBRG1 is not alternatively polyadenylated in ER- tumors (neither 3′US nor 3′UL). Globally, we checked that only 76 out of 958 HK ceRNA genes in ER- (7.9%) are either 3′US or 3′UL genes in tumors. This low overlap between our HK genes and 3′US genes implies that HK genes may not be directly related to growth-related functions (Curinha et al., 2014; Masamha et al., 2014) but contribute to tumorigenesis through 3′US-ceRNA. To further understand the impact of the repression on the ceRNA network, we compared the number of the ceRNA partners of these HK genes between normal and tumor. Previously, we showed that 3′US genes will break their relationship with the ceRNA partners (Park et al., 2018b). Since the ceRNA relationship changes, either loss or gain, could propagate to neighboring ceRNA relationships (Park et al., 2018a), the repression of HK genes should break the ceRNA relationship not only with 3′US genes but also with other ceRNA partners. Consistent to the expectation, 727 3′US HK ceRNA partners lost higher ratios of the ceRNA partners in tumor (Figure 4D). We found a similar trend of HK gene repression in ER+ breast cancer when connected to 3′US genes (Supplementary Table 6).

**Figure 5:**
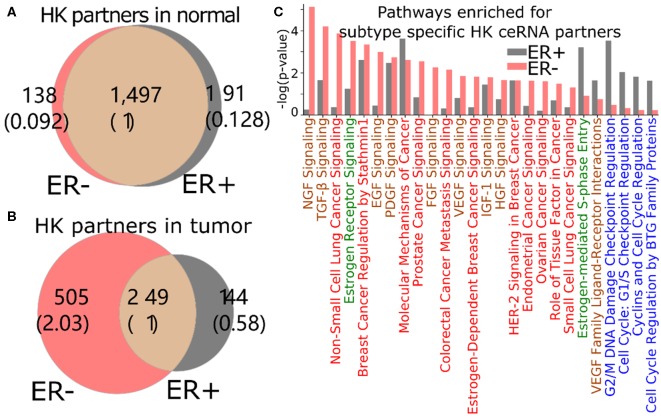
3′US disrupts the ceRNA relationship of HK genes for ER- specific growth. Number of HK ceRNA partners unique and common to ER- and ER+ normal **(A)** and tumor **(B)** ceRNA networks. The numbers in parentheses are normalized to the number of genes shared between tumor and normal. **(C)** IPA canonical pathways significantly (*P* < 0.01) enriched for ER+ and ER- specific HK ceRNA partners. The pathways are color-coded by keyword: “cancer” in red, “GF” in brown, “estrogen” in green, and “cell cycle” in blue.

The loss of HK ceRNA partners naturally reduces the high overlap of HK genes between ER+ and ER- (Figure 5A), resulting into 505 and 144 HK genes that are ceRNA partners of 3′US genes unique in ER- and ER+ tumor (ER- and ER+ HK ceRNA partners), respectively (Figure 5B). While it is known that cell growth and cell cycle regulations are different in the subtypes (Abba et al., 2005; Alles et al., 2009; Zhou et al., 2013), we found that the 505 ER- HK ceRNA partners are enriched for cell growth-related and cell cycle-related IPA pathways (Figure 5C, Supplementary Table 3). First, they are enriched for pathways associated to growth factor (with keyword “GF”). EGF (*P* = 10^−2.99^) especially activates cell cycle progression in ER- tumors (Biswas et al., 2000), and the expression of VEGF (*P* = 10^−2.42^) is associated to ER- tumors (Fuckar et al., 2006). Also, both EGF and VEGF are suspected to proliferate ER- tumors when estrogen cannot sustain them (Fuckar et al., 2006). Second, cell cycle pathways are enriched for ER+ specific HK ceRNA partners, suggesting that ER-regulated cell cycle (Paruthiyil et al., 2004; Javanmoghadam et al., 2016) differentiates ER+ and ER- cancer partially at the ceRNA level. Since the regulation of the cell cycle, G1- and S-phase and their transition ratio, is especially crucial for ER+ tumor's proliferation [reviewed in (Foster et al., 2002)], it is interesting that the cell cycle regulation pathways for various phases (G1/S or G2/M) of various mediators (estrogen or cyclins) are enriched with 144 ER+ HK ceRNA partners. Third, considering that the enrichment analysis was for the disjoint sets of genes (505 unique to ER- and 144 unique to ER+), it is interesting that these unique HK ceRNA partners are commonly significantly enriched for some “cancer” pathways, e.g., “molecular mechanisms of cancer,” showing that the HK ceRNAs are involved in cancer mechanisms equally significantly but in a subtype-specific fashion.

### 3′US Represses Housekeeping Genes to Promote Tumor Growth

**Figure 6:**
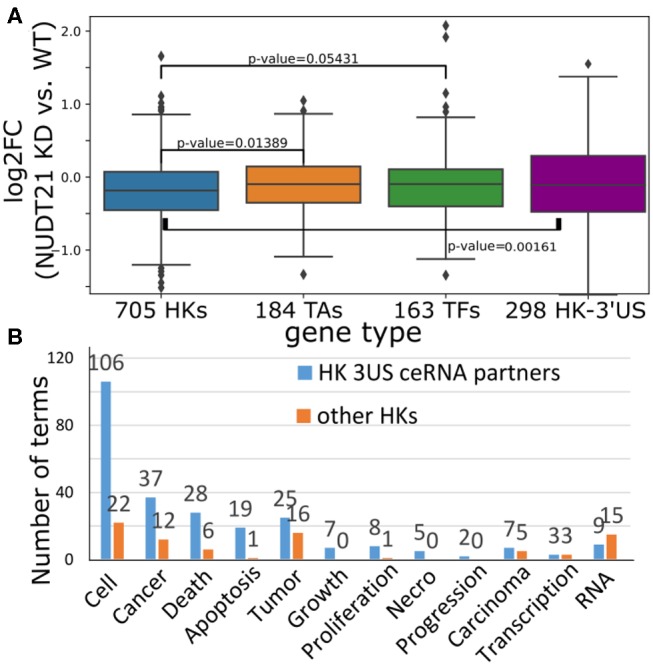
**(A)** Log_2_ fold change (NUDT21 KD vs. WT) of 705 HK, 184 TA, and 163 TF genes that are (potential) ceRNA partners of 3′US genes. Log_2_ fold change of 298 HK genes that are 3′US genes is displayed on the rightmost box. **(B)** Number of terms with the keyword indicated on the x-axis. Numbers on the bar represent the actual number of terms.

To gain insights into the cause-and-effect relationship from 3′US-mediated HK gene repression to tumorigenesis, we revisited a previous study (Masamha et al., 2014; Park et al., 2018b) in which 3′US-ceRNA effect promotes tumorigenesis in NUDT21 knockdown (KD) in HeLa cells and glioblastoma [data available in GSE42420 (Hammond et al., 2010) and GSE78198 (Ala et al., 2013)]. First, we chose 11,431 genes that are expressed in the experiment data (average FPKM >1). Among them, we further chose 4,430 genes that would work as miRNA sponges (>5 miRNA binding sites). To identify ceRNA relationship with the genes, we will solely use the significance of miRNA binding site overlap (FDR <0.05) since the other criteria for the ceRNA identification, co-expression, cannot be effectively estimated from two replicates of NUDT21 KD experiments. In this way, we identified 860 3′US genes and 2,449 of their ceRNA partners. Among these 3′US ceRNA partners, a significant portion of them (705, 28.8%) are HK genes, while 184 are TA and 163 are TF genes. It is especially interesting to note that HK genes in the network are only either 3′US genes (*n* = 298) or 3′US ceRNA partners (*n* = 705). On the other hand, almost half of the TA and TF genes in the network are not connected with 3′US genes [149 of 333 (44.7%) and 147 of 310 (47.4%) for TA and TF, respectively], showing that HK genes can be a major target of 3′US ceRNA effect. Based on our previous finding that 3′US represses the ceRNA partners in tumor (Park et al., 2018b), we further checked the repression of HK genes in NUDT21 KD. A total of 705 HK genes that are 3′US ceRNA partners are more repressed than TA and TF genes or than 298 HK 3′US genes in the network (Figure 6A; *P* = 0.01 and 0.05, 0.002, respectively). These results confirm that HK genes are repressed in the tumorigenic process that 3′US-ceRNA effect promotes (Park et al., 2018b).

To assess the impact of this repression on tumor growth, we further conducted IPA analysis on 705 HK 3′US ceRNA partners in comparison to the other 2,410 HK genes not in the network. First, although there are much less HK 3′US ceRNA partners than the other HK genes, they are enriched for more IPA diseases and functions terms (Supplementary Table 4). While the IPA analysis gives N/A for the terms that are so lowly enriched that cannot be estimated, HK 3′US ceRNA partners have 581 terms with N/A value and HK genes not in the network have 693 terms with N/A value. Furthermore, we replaced the N/A values with the minimum value and compared the *p*-values in HK 3′US ceRNA partners vs. the other HK genes. This comparison shows that more terms are significantly (*P* < 0.01) enriched for HK 3′US ceRNA partners (254 terms with better *p*-values for HK 3′US ceRNA partners and 141 for the other HK genes). This trend is more pronounced for the terms that are important for cancer. For example, IPA terms with keywords “cell,” “cancer (or tumor),” “apoptosis (death, or necro),” and “growth (proliferation, or progression)” are significantly (*P* < 2.2e^−16^) more enriched in the HK 3′US ceRNA partners, while certain terms for general biological processes such as “RNA” are enriched in the other HK genes (Figure 6B). While this analysis does not support our hypothesis as a whole, it demonstrates a potential role of HK gene repression in a tumorigenesis process with HeLa as a model system. It follows that the ER- specific tumor progression is attributable to the repression of different HK genes.

## Discussion

To investigate the role of 3′US-ceRNA effect (Park et al., 2018b) for ER- vs. ER+ breast tumors, we built the ceRNA networks that are comparable to each other subtype by addressing the bias due to the different numbers of samples (72 for ER+ and 20 for ER- in TCGA). A fair comparison of the networks suggests that 3′US disrupts the ceRNA network for ER- tumors' aggressive phenotypes. Furthermore, we revealed a role of 3′US-ceRNA effect on HK genes. In the cancer context, the potential for being ceRNA was identified for mRNAs [e.g., (Tay et al., 2011)] as well as long non-coding RNAs [*e.g.*, (Tuersong et al., 2019)] and pseudogenes [e.g., (Wei et al., 2017)]. Among mRNAs, it has been shown that TA genes and TF genes heavily contribute to ceRNA regulation (Sumazin et al., 2011). While reaffirming the high contribution (and thus high potential of biological function) of the TA and TF genes to breast cancer ceRNA networks, we further found the high contribution of HK genes. HK genes were reported as stable “control” genes for miRNA sponge effect [e.g., (Bouhaddioui et al., 2014)], indirectly supporting our novel findings. By analyzing TCGA breast cancer and reanalyzing an experimental data, we found more direct supports for their roles to ceRNA.

Further analyses show that 3′US disrupts the ceRNA crosstalk of HK genes in a subtype-specific fashion. First, we showed that a subset of HK genes is *trans* target of 3′US-ceRNA effect (sponge HK genes) enriched in important pathways in association to ER-'s aggressive phenotype. Since they are much less than the other HK genes in number (*e*. *g*., 705 3′US ceRNA HK genes vs. 2,401 HK genes in the NUDT21 KD experiment), our definition may shed novel insights into identifying another set of biomarkers indicating tumor progression.

In network analysis, a network of interest is often compared to a reference network. However, if the networks are built from different numbers of samples, the comparison will be biased due to the sample size difference (Supplementary Figure 3). With the assumption that normal samples should have similar molecular dynamics, we found the subtype-specific cutoff values for normal ceRNA networks. Then, we constructed ER+ and ER- tumor ceRNA networks (two-step pairwise normalization method). As the resulting ceRNA networks facilitate novel discoveries on the subtype-specific 3′US-ceRNA effect, we expect that the two-step pairwise normalization method can further help normalize biological networks built with the different numbers of samples if the matched normal samples are available.

We note that this normalization method can help further identify the genes playing important roles in a subtype-specific fashion. For example, we used the KS test to compare the eigenvalue distribution of the Laplacian matrix of the two networks, ER+ and ER- ceRNA networks. The eigenvalue distribution is a set of eigenvalues, each representing a temporal snapshot of the network (Gera et al., 2018). Since *K*_1,2_ in Equation 4 represents the snapshot point at which the topology of the two networks is most apart, the edges appearing at that time point strongly differentiate the two subtypes in the ceRNA level. In that sense, genes in the edges can be further investigated for their roles in each of the subtypes. Also, the resulting networks, the comparable ceRNA networks of ER+ and ER– breast tumors, can further help identify important genes for specific functions in the subtypes. Biological network analysis techniques were used to identify the genes playing important roles in the ceRNA network (Sumazin et al., 2011; Cheng et al., 2015). To identify such genes for ER- tumor, the samples need to be compared with ER+ in our context. In that sense, we can build a differential network (ER– vs. ER+) based on the comparable ceRNA networks. Then, since hub genes in the differential network would facilitate the ceRNA regulation of many genes only for a specific subtype, e.g., ER- breast tumor, they would be good candidates for important functions specific to the ER- tumors. We can further identify those for specific functions based on the gene sets defined for the functions, e.g., gene ontology (The Gene Ontology Consortium, 2018). Our study showed the distinct 3′US-ceRNA dynamics between the ER+ and the ER- groups of tumor samples. Although ER status is an important clinical variable (Hammond et al., 2010), it is important to note that the two groups do not directly represent further clinical subtypes of breast cancers, such as HER2+ or triple-negative. Thus, to reveal a further clinical relevance of 3′US-ceRNA dynamics, more study is warranted in the clinical subtypes within each group.

## Data Availability Statement

All datasets generated for this study are included in the article/Supplementary Material.

## Author Contributions

HP conceived the project and designed the experiments. ZF performed the data analysis with the help from YB. SK conducted statistical tests. HP wrote the manuscript with input from BD and SK.

## Conflict of Interest

The authors declare that the research was conducted in the absence of any commercial or financial relationships that could be construed as a potential conflict of interest.

## References

[B1] Abba M. C. Hu Y. Sun H. Drake J. A. Gaddis S. Baggerly K.. (2005). Gene expression signature of estrogen receptor α status in breast cancer. BMC Genomics 6, 1–13. 10.1186/1471-2164-6-37 15762987 PMC555753

[B2] Ala U. Karreth F. A. Bosia C. Pagnani A. Taulli R. Léopold V.. (2013). Integrated transcriptional and competitive endogenous RNA networks are cross-regulated in permissive molecular environments. Proc. Natl. Acad. Sci. U.S.A. 110, 7154–7159. 10.1073/pnas.1222509110 23536298 PMC3645534

[B3] Alles M. C. Gardiner-Garden M. Nott D. J. Wang Y. Foekens J. A. Sutherland R. L.. (2009). Meta-analysis and gene set enrichment relative to ER status reveal elevated activity of MYC and E2F in the ‘basal’ breast cancer subgroup. PLoS ONE 4:e4710. 10.1371/journal.pone.0004710 19270750 PMC2650420

[B4] Altay G. Asim M. Markowetz F. Neal D. E. (2011). Differential C3NET reveals disease networks of direct physical interactions. BMC Bioinformatics 12:296. 10.1186/1471-2105-12-296 21777411 PMC3156794

[B5] Bailey T. L. Boden M. Buske F. A. Frith M. Grant C. E. Clementi L.. (2009). MEME suite: tools for motif discovery and searching. Nucleic Acids Res. 37, 202–208. 10.1093/nar/gkp335 19458158 PMC2703892

[B6] Bastos E. P. Brentani H. Pasini F. S. Silva A. R. Torres C. H. Puga R. D.. (2014). MicroRNAs discriminate familial from sporadic Non-BRCA1/2 breast carcinoma arising in patients ≤35 years. PLoS ONE 9:e101656. 10.1371/journal.pone.0101656 25006670 PMC4090167

[B7] Biswas D. K. Cruz A. P. Gansberger E. Pardee A. B. (2000). Epidermal growth factor-induced nuclear factor kappa b activation: a major pathway of cell-cycle progression in estrogen-receptor negative breast cancer cells. Proc. Natl. Acad. Sci. U.S.A. 97, 8542–8547. 10.1073/pnas.97.15.8542 10900013 PMC26984

[B8] Bouhaddioui W. Provost P. R. Tremblay Y. (2014). Identification of most stable endogenous control genes for MicroRNA quantification in the developing mouse lung. PLoS ONE 9:e111855. 10.1371/journal.pone.0111855 25368994 PMC4219792

[B9] Busuttil R. A. Rubio M. Dollé M. E. Campisi J. Vijg J. (2003). Oxygen accelerates the accumulation of mutations during the senescence and immortalization of murine cells in culture. Aging Cell 2, 287–294. 10.1046/j.1474-9728.2003.00066.x 14677631

[B10] Chawla K. Tripathi S. Thommesen L. Lægreid A. Kuiper M. (2013). TFcheckpoint: a curated compendium of specific DNA-binding RNA polymerase II transcription factors. Bioinformatics 29, 2519–2520. 10.1093/bioinformatics/btt432 23933972

[B11] Chen H. Li C. Peng X. Zhou Z. Weinstein J. N. The cancer genome atlas research network. (2018). A pan-cancer analysis of enhancer expression in nearly 9000 patient samples. Cell 173, 386–399.e12. 10.1016/j.cell.2018.03.027 29625054 PMC5890960

[B12] Chen M. Lyu G. Han M. Nie H. Shen T. Chen W.. (2018). 3′ UTR lengthening as a novel mechanism in regulating cellular senescence. Genome Res. 285−294. 10.1101/gr.224451.117 29440281 PMC5848608

[B13] Cheng D. L. Xiang Y. Y. Ji L. J. Lu X. J. (2015). Competing endogenous RNA interplay in cancer : mechanism, methodology, and perspectives. Tumour Biol. 36, 479–488. 10.1007/s13277-015-3093-z 25604144

[B14] Curinha A. Oliveira Braz S. Pereira-Castro I. Cruz A. Moreira A. (2014). Implications of polyadenylation in health and disease. Nucleus 5, 508–519. 10.4161/nucl.36360 25484187 PMC4615197

[B15] Curmi P. A. Noguès C. Lachkar S. Carelle N. Gonthier M. P. Sobel A.. (2000). Overexpression of stathmin in breast carcinomas points out to highly proliferative tumours. Br. Cancer J. 82, 142–150. 10.1054/bjoc.1999.0891 10638981 PMC2363189

[B16] Dalgiç E. Konu Ö. Öz Z. S. Chan C. (2019). Lower connectivity of tumor coexpression networks is not specific to cancer. In silico Biol. 13, 41–53. 10.3233/ISB-190472 31156157 PMC6597990

[B17] Davoli T. Xu A. W. Mengwasser K. E. Sack L. M. Yoon J. C. Park P. J.. (2013). Cumulative haploinsufficiency and triplosensitivity drive aneuploidy patterns and shape the cancer genome. Cell 155, 948–962. 10.1016/j.cell.2013.10.011 24183448 PMC3891052

[B18] Dimri G. P. Lee X. Basile G. Acosta M. Scott G. Roskelley C.. (1995). A biomarker that identifies senescent human cells in culture and in aging skin *in vivo*. Proc. Natl. Acad. Sci. U.S.A. 92, 9363–9367. 10.1073/pnas.92.20.9363 7568133 PMC40985

[B19] Dvinge H. Git A. Gräf S. Salmon-Divon M. Curtis C. Sottoriva A.. (2013). The shaping and functional consequences of the microRNA landscape in breast cancer. Nature 497, 378–382. 10.1038/nature12108 23644459

[B20] Eisenberg E. Levanon E. Y. (2003). Human housekeeping genes are compact. Trends Genet. 19, 362–365. 10.1016/S0168-9525(03)00140-9 12850439

[B21] Eisenberg E. Levanon E. Y. (2013). Human housekeeping genes, revisited. Trends Genet. 29, 569–574. 10.1016/j.tig.2013.05.010 23810203

[B22] Foster J. S. Henley D. C. Bukovsky A. Seth P. Wimalasena J. (2002). Multifaceted regulation of cell cycle progression by estrogen: regulation of cdk inhibitors and Cdc25A independent of cyclin D1-Cdk4 function. Mol. Cell. Biol. 21, 794–810. 10.1128/MCB.21.3.794-810.2001 11154267 PMC86671

[B23] Friedel C. C. Zimmer R. (2006). Inferring topology from clustering coefficients in protein-protein interaction networks. BMC Bioinformatics 15, 1–15. 10.1186/1471-2105-7-519 PMC1716184 17137490

[B24] Fuckar D. Dekanić A. Stifter S. Mustać E. Krstulja M. Dobrila F.. (2006). VEGF expression is associated with negative estrogen receptor status in patients with breast cancer. Int. J. Surg. Pathol. 14, 49–55. 10.1177/106689690601400109 16501835

[B25] Gera R. Alonso L. Crawford B. House J. Mendez-Bermudez J. A. Knuth T.. (2018). Identifying network structure similarity using spectral graph theory. Appl. Netw. Sci. 3:2. 10.1007/s41109-017-0042-3 30839726 PMC6214265

[B26] Goldman M. Craft B. Swatloski T. Ellrott K. Cline M. Diekhans M.. (2013). The UCSC cancer genomics browser: update 2013. Nucleic Acids Res. 41, D949–D954. 10.1093/nar/gks1008 23109555 PMC3531186

[B27] Hamilton M. P. Rajapakshe K. Hartig S. M. Reva B. McLellan M. D. Kandoth C.. (2013). Identification of a pan-cancer oncogenic microRNA superfamily anchored by a central core seed motif. Nat. Commun. 4:2730. 10.1038/ncomms3730 24220575 PMC3868236

[B28] Hammond M. E. Hayes D. F. Dowsett M. Allred D. C. Hagerty K. L. Badve S.. (2010). American Society of Clinical Oncology/College of American Pathologists guideline recommendations for immunohistochemical testing of estrogen and progesterone receptors in breast cancer (unabridged version). Arch. Pathol. Lab. Med. 134, e48–72. 10.1043/1543-2165-134.7.e48 20586616

[B29] Hsu S. D. Tseng Y. T. Shrestha S. Lin Y. L. Khaleel A. Chou C. H.. (2014). miRTarBase update 2014: an information resource for experimentally validated miRNA-target interactions. Nucleic Acids Res. 42, D78–D85. 10.1093/nar/gkt1266 24304892 PMC3965058

[B30] Javanmoghadam S. Weihua Z. Hunt K. K. Keyomarsi K. (2016). Estrogen receptor alpha is cell cycle-regulated and regulates the cell cycle in a ligand-dependent fashion. Cell Cycle 15 12, 1579–1590. 10.1080/15384101.2016.1166327 PMC4934046 27049344

[B31] Kim S. Bai Y. Fan Z. Diergaarde B. Tseng G. C. Park H. J. (2019). Alternative polyadenylation regulates patient-specific tumor growth by individualizing the MicroRNA target site landscape. bioRxiv, 601518 10.1101/601518

[B32] Krishnan K. Steptoe A. L. Martin H. C. Pattabiraman D. R. Nones K. Waddell N.. (2013). miR-139-5p is a regulator of metastatic pathways in breast cancer. RNA 19, 1767–1780. 10.1261/rna.042143.113 24158791 PMC3884652

[B33] Lee J. Y. Yeh I. Park J. Y. Tian B. (2007). PolyA_DB 2: mRNA polyadenylation sites in vertebrate genes. Nucleic Acids Res. 35 (suppl_1), D165–D168. 10.1093/nar/gkl870 17202160 PMC1899096

[B34] Lewis B. P. Burge C. B. Bartel D. P. (2005). Conserved seed pairing, often flanked by adenosines, indicates that thousands of human genes are microRNA targets. Cell 120, 15–20. 10.1016/j.cell.2004.12.035 15652477

[B35] López-Otín C. Blasco M. Partridge A. L. Serrano M. Kroemer G. (2013). The hallmarks of aging. Cell 153, 1194–1217. 10.1016/j.cell.2013.05.039 23746838 PMC3836174

[B36] Masamha C. P. Xia Z. Yang J. Albrecht T. R. Li M. Shyu A. B.. (2014). CFIm25 links alternative polyadenylation to glioblastoma tumour suppression. Nature 510, 412–416. 10.1038/nature13261 24814343 PMC4128630

[B37] Mayr C. Bartel D. P. (2009). Widespread shortening of 3'UTRs by alternative cleavage and polyadenylation activates oncogenes in cancer cells. Cell 138, 673–684. 10.1016/j.cell.2009.06.016 19703394 PMC2819821

[B38] Muñoz-Espín D. Serrano M. (2014). Cellular senescence: from physiology to pathology. Nat. Rev. Mol. Cell Biol. 15, 482–496. 10.1038/nrm3823 24954210

[B39] Obayashi S. Horiguchi J. Higuchi T. Katayama A. Handa T. Altan B.. (2017). Stathmin1 expression is associated with aggressive phenotypes and cancer stem cell marker expression in breast cancer patients. Int. J. Oncol. 51, 781–790. 10.3892/ijo.2017.4085 28766688 PMC5564402

[B40] Papadopoulos G. L. Reczko M. Simossis V. A. Sethupathy P. Hatzigeorgiou A. G. (2009). The database of experimentally supported targets: a functional update of TarBase. Nucleic Acids Res. 37, D155–D158. 10.1093/nar/gkn809 18957447 PMC2686456

[B41] Park H. J. Ji P. Kim S. Xia Z. Rodriguez B. Li L.. (2018b). 3′ UTR shortening represses tumor-suppressor genes in trans by disrupting ceRNA crosstalk. Nat. Genet. 50, 783–789. 10.1038/s41588-018-0118-8 29785014 PMC6689271

[B42] Park H. J. Kim S. Li W. (2018a). Model-based analysis of competing- endogenous pathways (MACPath) in human cancers. PLoS Comput. Biol. 22:e1006074 10.1371/journal.pcbi.1006074 PMC5882149 29565967

[B43] Paruthiyil S. Parmar H. Kerekatte V. Cunha G. R. Firestone G. L. Leitman D. C. (2004). Estrogen receptor? inhibits human breast cancer cell proliferation and tumor formation by causing a G 2 cell cycle arrest. Cancer Res. 64, 423–428. 10.1158/0008-5472.CAN-03-2446 14729654

[B44] Perou C. M. Sørlie T. Eisen M. B. van de Rijn M. Jeffrey S. S. Rees C. A.. (2002). Molecular portraits of human breast tumours. Nature 406, 747–752. 10.1038/35021093 10963602

[B45] Salmena L. Poliseno L. Tay Y. Kats L. Pandolfi P. P. (2011). A ceRNA hypothesis: the rosetta stone of a hidden RNA language? Cell 146, 353–358. 10.1016/j.cell.2011.07.014 21802130 PMC3235919

[B46] Sheikh M. Garcia M Pujol P Fontana JA Rochefort H. (1994). Why are estrogen-receptor-negative breast cancers more aggressivet. Invasion Metastasis 14, 329–336. 7657526

[B47] Sumazin P. Yang X. Chiu H. S. Chung W. J. Iyer A. Llobet-Navas D.. (2011). An extensive microRNA-mediated network of RNA-RNA interactions regulates established oncogenic pathways in glioblastoma. Cell 147, 370–381. 10.1016/j.cell.2011.09.041 22000015 PMC3214599

[B48] Tay Y. Kats L. Salmena L. Weiss D. Tan S. M. Ala U.. (2011). Coding-independent regulation of the tumor suppressor PTEN by competing endogenous mRNAs. Cell 147, 344–357. 10.1016/j.cell.2011.09.029 22000013 PMC3235920

[B49] Tay Y. Rinn J. Pandolfi P. P. (2014). The multilayered complexity of ceRNA crosstalk and competition. Nature 505, 344–352. 10.1038/nature12986 24429633 PMC4113481

[B50] The Gene Ontology Consortium (2018). The Gene Ontology Resource: 20 years and still GOing strong. Nucleic Acids Res. 47, D330–D338. 10.1093/nar/gky1055 PMC6323945 30395331

[B51] Tsutsui S. Ohno S. Murakami S. Hachitanda Y. Oda S. (2002). Prognostic value of epidermal growth factor receptor (EGFR) and its relationship to the estrogen receptor status in 1029 patients with breast cancer. Breast Cancer Res. Treat. 71, 67–75. 10.1023/A:1013397232011 11859875

[B52] Tuersong T. Li L. Abulaiti Z. Feng S. (2019). Comprehensive analysis of the aberrantly expressed lncRNA-associated ceRNA network in breast cancer. Mol. Med. Rep. 19, 4697–4710. 10.3892/mmr.2019.10165 31059025 PMC6522813

[B53] Vaught D. Brantley-Sieders D. M. Chen J. (2008). Eph receptors in breast cancer: roles in tumor promotion and tumor suppression. Breast Cancer Res. 10:217. 10.1186/bcr2207 19144211 PMC2656900

[B54] Wei Y. Chang Z. Wu C. Zhu Y. Li K. Xu Y. (2017). Identification of potential cancer-related pseudogenes in lung adenocarcinoma based on ceRNA hypothesis. Oncotarget 8, 59036–59047. 10.18632/oncotarget.19933 28938616 PMC5601712

[B55] Xia Z. Donehower L. A. Cooper T. A. Neilson J. R. Wheeler D. A. Wagner E. J.. (2014). Dynamic analyses of alternative polyadenylation from RNA- Seq reveal landscape of 3' UTR usage across 7 tumor types. Nat. Commun. 5:5274. 10.1038/ncomms6274 25409906 PMC4467577

[B56] Xiang Y. Ye Y. Lou Y. Yang Y. Cai C. Zhang Z.. (2018). Comprehensive characterization of alternative polyadenylation in human cancer. J. Natl. Cancer Inst. 110, 1–11. 10.1093/jnci/djx223 29106591 PMC6059203

[B57] Xiao F. Zuo Z. Cai G. Kang S. Gao X. Li T. (2009). miRecords: an integrated resource for microRNA-target interactions. Nucleic Acids Res. 37, 105–110. 10.1093/nar/gkn851 18996891 PMC2686554

[B58] Zhenjiang F. Kim S. Diergaarde B. Park H. J. (2019). 3′-UTR shortening disrupts ceRNA crosstalk of housekeeping genes resulting in subtype-specific breast cancer development. bioRxiv. 601526 10.1101/601526

[B59] Zhou X. Shi T. Li B. Zhang Y. Shen X. Li H.. (2013). Genes dysregulated to different extent or oppositely in estrogen receptor-positive and estrogen receptor-negative breast cancers. PLoS ONE 8:e70017. 10.1371/journal.pone.0070017 23875016 PMC3715479

