# Supplementary material for: 3′-UTR Shortening Contributes to Subtype-Specific Cancer Growth by Breaking Stable ceRNA Crosstalk of Housekeeping Genes
Source: Front Bioeng Biotechnol. 2020 Apr 29;8:334. doi: 10.3389/fbioe.2020.00334 (PMC7201092; doi:10.3389/fbioe.2020.00334)
Supplement: Supplementary file 2 [file Image_1.pdf]

Supplementary Figures

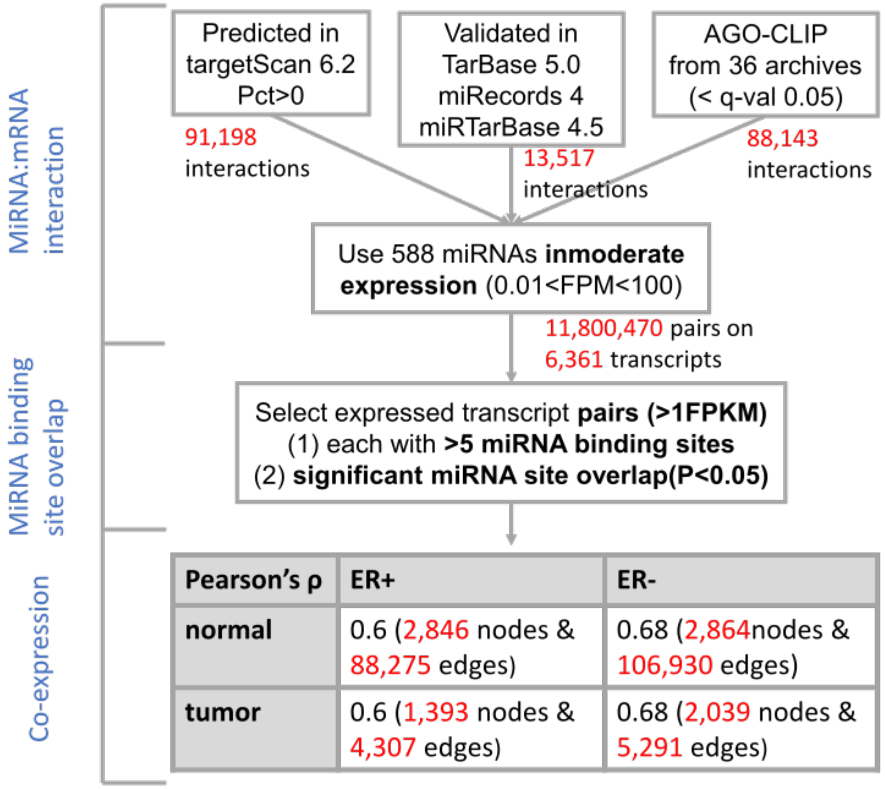

S. Figure 1. Workflow for the ceRNA network construction for the TCGA breast tumor and the matched normal samples of ER+ and ER- subtypes

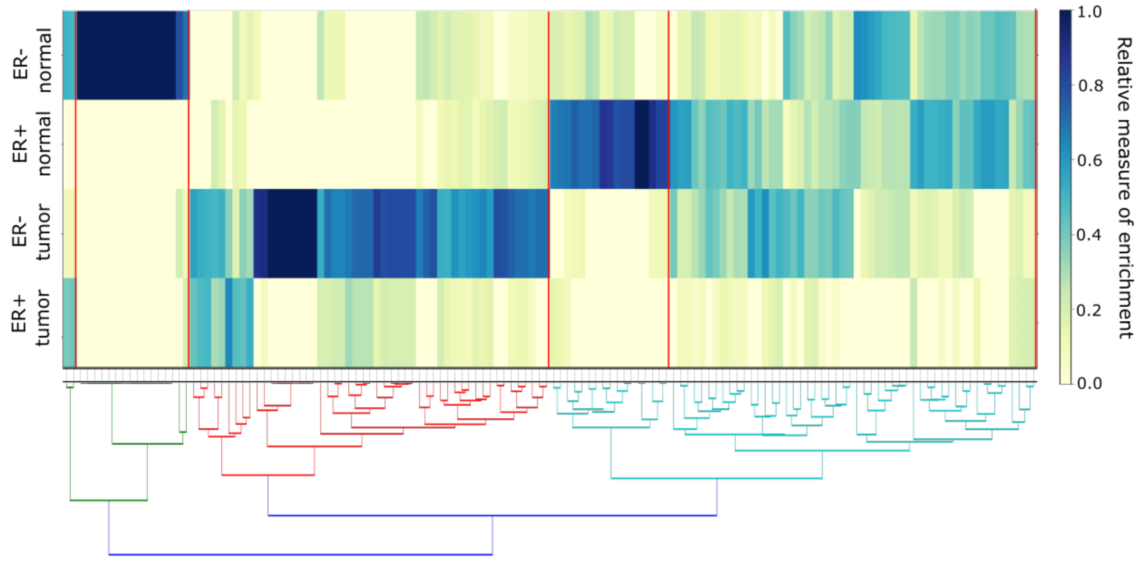

S. Figure 2. IPA pathways enriched for the recurrent 3′UL and 3′US genes in ER- and ER+. Colors represent enrichment of each pathway (column) for each class of genes (row). The red lines in the heatmap cut the pathways into 5 clusters in accordance with the dendrogram drawn on the bottom.

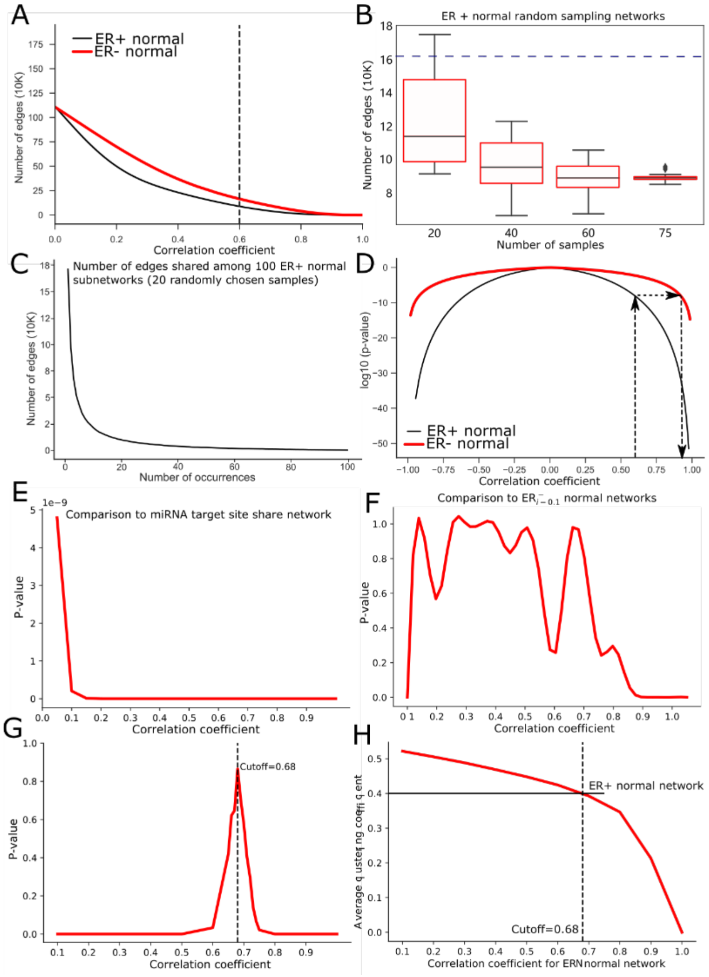

S. Figure 3. Two-step Pairwise Normalization of ER+ and ER- ceRNA network. (A) Number of edges in the ceRNA networks by the correlation coefficient cutoff (black and red line for ER+ and ER- normal networks, respectively). (B) Number of edges in 100 networks built from a subset of ER+ normal samples in different size. Blue dotted line indicates the number of edges of ER- normal network whose sample size is 20 (160,687) (C) The number of edges shared among 100 ER+ normal samples, where each of them was built by using 20 randomly chosen samples. (D) Statistical significance (p-value) achievable by using different correlation coefficient cutoff values for ER+ (black) and ER- (red) samples. Statistical significance for a correlation coefficient cutoff value is described in Methods.. (E) Comparison of ER- normal network with the miRNA target site share network to by correlation cutoff value (see Methods). (F) Comparison of ER- normal network with that of the previous correlation cutoff value in the stepwise increase (see Methods). (G). Significance of topological similarity (y-axis) of ER+ normal network with ER- normal ceRNA networks built by different cutoff values (x-axis). The bigger the p-value is, the more similar the two networks are (see Methods)[32]. (H) Comparison of the ER+ normal reference network with ER- normal ceRNA networks built by different correlation cutoff values in the average clustering coefficient..

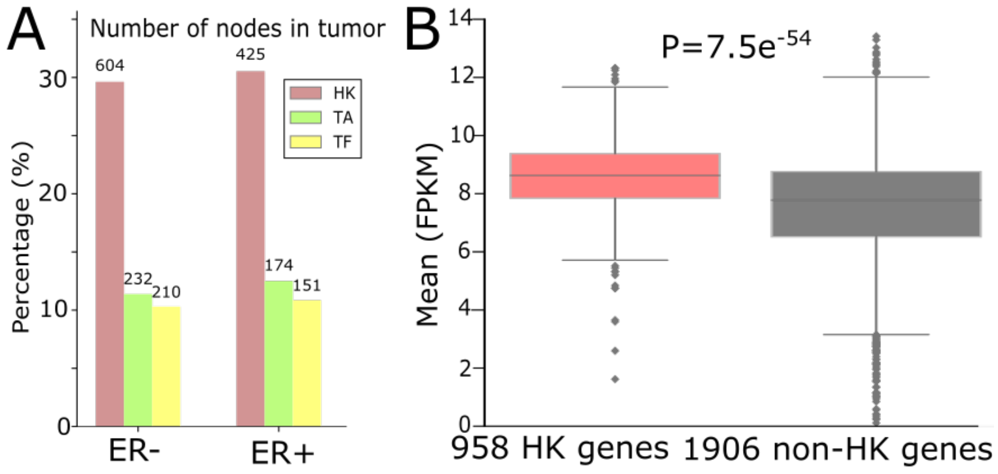

S. Figure 4. (A) Number (and the percentage to the total number of nodes in tumor networks) of HK genes and other important classes of genes in ER+ and ER- normal ceRNA networks. (B) Average gene expression values of 958 HK genes and 1,906 non-HK genes in the ER+ and ER- normal samples.
